# Evaluation of [^11^C]oseltamivir uptake into the brain during immune activation by systemic polyinosine-polycytidylic acid injection: a quantitative PET study using juvenile monkey models of viral infection

**DOI:** 10.1186/s13550-014-0024-8

**Published:** 2014-07-02

**Authors:** Chie Seki, Arata Oh-Nishi, Yuji Nagai, Takafumi Minamimoto, Shigeru Obayashi, Makoto Higuchi, Makoto Takei, Kenji Furutsuka, Takehito Ito, Ming-Rong Zhang, Hiroshi Ito, Mototsugu Ito, Sumito Ito, Hiroyuki Kusuhara, Yuichi Sugiyama, Tetsuya Suhara

**Affiliations:** 1 Molecular Imaging Center, National Institute of Radiological Sciences, 4-9-1 Anagawa, Inage-ku 265-8555, Chiba, Japan; 2 Laboratory of Molecular Pharmacokinetics, Graduate School of Pharmaceutical Sciences, The University of Tokyo, 7-3-1 Hongo, Bunkyo-ku 133-0033, Tokyo, Japan; 3 Sugiyama Laboratory, RIKEN Innovation Center, Research Cluster for Innovation, RIKEN, 1-6, Suehiro-cho, Tsurumi-ku, Yokohama 230-0045, Kanagawa, Japan

**Keywords:** Positron emission tomography, Oseltamivir, Brain, Monkey, Poly I:C

## Abstract

**Background:**

Abnormal behaviors of young patients after taking the anti-influenza agent oseltamivir (Tamiflu®, F. Hoffmann-La Roche, Ltd., Basel, Switzerland) have been suspected as neuropsychiatric adverse events (NPAEs). Immune response to viral infection is suspected to cause elevation of drug concentration in the brain of adolescents. In the present study, the effect of innate immune activation on the brain uptake of [^11^C]oseltamivir was quantitatively evaluated in juvenile monkeys.

**Methods:**

Three 2-year-old monkeys underwent positron emission tomography (PET) scans at baseline and immune-activated conditions. Both scans were conducted under pre-dosing of clinically relevant oseltamivir. The immune activation condition was induced by the intravenous administration of polyinosine-polycytidylic acid (poly I:C). Dynamic [^11^C]oseltamivir PET scan and serial arterial blood sampling were performed to obtain [^11^C]oseltamivir kinetics. Brain uptake of [^11^C]oseltamivr was evaluated by its normalized brain concentration, brain-to-plasma concentration ratio, and plasma-to-brain transfer rate. Plasma pro-inflammatory cytokine levels were also measured.

**Results:**

Plasma interleukin-6 was elevated after intravenous administration of poly I:C in all monkeys. Brain radioactivity was uniform both at baseline and under poly I:C treatment. The mean brain concentrations of [^11^C]oseltamivir were 0.0033 and 0.0035% ID/cm^3^ × kg, the mean brain-to-plasma concentration ratios were 0.58 and 0.65, and the plasma-to-brain transfer rates were 0.0047 and 0.0051 mL/min/cm^3^ for baseline and poly I:C treatment, respectively. Although these parameters were slightly changed by immune activation, the change was not notable.

**Conclusions:**

The brain uptake of [^11^C]oseltamivir was unchanged by poly I:C treatment in juvenile monkeys. This study demonstrated that the innate immune response similar to the immune activation of influenza would not notably change the brain concentration of oseltamivir in juvenile monkeys.

## Background

Oseltamivir (Tamiflu®, F. Hoffmann-La Roche, Ltd., Basel, Switzerland) is the orally active ester prodrug of the anti-influenza agent Ro 64-0802, a potent and selective viral neuraminidase inhibitor that is effective for the treatment of influenza A, B, and A (H1N1). Unusual neuropsychiatric events even including suicidal events in young patients taking oseltamivir have been reported in Japan [[1]]. Such incidents have been suspected as neuropsychiatric adverse events (NPAEs) of oseltamivir, although the underlying mechanisms have not yet been clarified.

Due to such issues, great efforts have been made to elucidate the factors affecting oseltamivir penetration into the central nervous system (CNS) in rodents and nonhuman primates. It was elucidated that P-glycoprotein (P-gp) at the blood–brain barrier (BBB) limits plasma oseltamivir penetration into the brain [[2],[3]]. Ro 64-0802 barely diffuses into the brain, and its high hydrophilicity (cLogP −0.97) was initially considered to be the reason [[3],[4]]. However, a later study using mice demonstrated that active efflux at the BBB mediated by organic anion transporter 3 (Oat3) and multidrug resistance protein 4 (Mrp4) also contributes to the low brain penetration [[5]]. In healthy adult humans, the cerebrospinal fluid/plasma ratio of oseltamivir and Ro 64-0802 were at most 2.1% and 3.5%, respectively, suggesting their low brain penetration [[6]]. To investigate the brain penetration in humans and nonhuman primates, [^11^C]oseltamivir and [^11^C]Ro 64-0802 were synthesized as positron emission tomography (PET) imaging probes [[4]]. Recent studies demonstrated that the BBB penetration of [^11^C]oseltamivir was slightly higher in adolescent monkeys than that in adults [[7],[8]].

NPAEs have occurred in young patients during relatively early phase after the onset of influenza-like symptoms [[9]]. Furthermore, it was reported that 30% of the abnormal behaviors of pediatric patients occurred within 2 h after the first use of oseltamivir [[9]], which is close to the time of reaching maximum plasma concentrations of oseltamivir and Ro 64-0802 [[6]]. It is possible that the immune response leads to the elevation of CNS oseltamivir concentration by increasing BBB permeability and/or plasma oseltamivir concentration. Influenza virus infection strongly activates the immune system and induces pro-inflammatory cytokines and chemokines systemically. Some in vitro studies have reported that pro-inflammatory cytokines alter the integrity of BBB or induce modulation of efflux transporters [[10]]. It is reported that the expression of human carboxylesterase 1 (CES1), responsible for conversion of oseltamivir to Ro 64-0802 [[11]], was suppressed by pro-inflammatory cytokine interleukin-6 (IL-6) in vitro [[12]]. This highlights the importance of investigating the effect of viral infection on the CNS exposure of oseltamivir and Ro 64-8082 under therapeutic dosage in vivo.

In the present study, we attempted to evaluate the alterations of CNS uptake of [^11^C]oseltamivir under similar immune activation of viral infection under therapeutic dose of oseltamivir medication in living juvenile monkeys using PET. Immune activation models of *Rhesus* monkeys have been developed using synthetic double-stranded RNA, polyinosine-polycytidylic acid (poly I:C), which induces increasing IL-6 production in the blood [[13]]. We used an immune activation model in juvenile monkeys where poly I:C was administered as an experimental model of viral infection. Stimulation by the systemic administration of poly I:C is similar to that by viral infection upstream of the innate immune response, such as the induction of pro-inflammatory cytokines [[14]] and interferon in monkeys [[15]].

The advantages of the use of monkeys over rodents are the following: (1) their CNS developmental changes are similar to those in humans; (2) oseltamivir is converted to Ro 64-8082 by hepatic CES in monkeys as in humans, whereas serum CES activity is high in rodents [[3]]; and (3) monkeys are capable of undergoing serial blood samplings required for estimation of BBB permeability. To achieve therapeutically relevant plasma concentrations of oseltamivir and Ro 64-0802 during PET scan, unlabeled oseltamivir was administered because the dose of [^11^C]oseltamivir for PET imaging was approximately 10 μg/kg. This study is the first to report the impact of immune activation on oseltamivir brain uptake in living juvenile monkeys under therapeutic oseltamivir dosage.

## Methods

### Animals

Three 2-year-old monkeys (*Macaca fuscata*, one male and two females; M176, 29 months, 4.0 kg; F175, 32 months, 4.9 kg; and F174, 33 months, 3.9 kg) were used. One 3-year-old male monkey (*Macaca mulatta*) was used to obtain MRI images and baseline [^11^C]oseltamivir PET images for a preliminary experiment. The animals were kept under pentobarbital anesthesia (i.v. 20 to 25 mg/kg) and warmed by heating pad. Electrocardiogram and body temperature, only during poly I:C treatment condition, were taken with a vital monitoring system (BP-88 V, OMRON Co., Ltd., Kyoto, Japan). All the monkeys fully recovered to normal conditions after each experiment. The monkeys were maintained and handled in accordance with the guidelines published in the National Institutes of Health (NIH) Guide for the Care and Use of Laboratory Animals (NIH, publication no. 86-23, revised 1987) and the guidelines of the National Institute of Radiological Sciences (NIRS). The present study was approved by the Animal Ethics Committee of NIRS, Chiba, Japan.

### PET study

**Figure 1:**
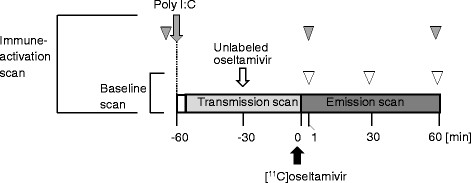
Schematic diagram of the experimental protocol.

The outline of the experimental protocol is summarized in Figure 1. In each animal, a set of scans under baseline and immune activation conditions was performed in this order, with an interscan interval of 4 weeks or longer for recovery from blood loss due to arterial blood samplings.

#### Unlabeled oseltamivir loading

Oseltamivir phosphate (3.2 ± 0.1 mg/kg, Sequoia Research Products, Pangbourne, UK) dissolved in sterilized distilled water was intravenously administered 30 min before the initiation of the PET scan (closed arrow) to maintain equivalent plasma drug levels under the highest single therapeutic dose during the PET scan (open arrow). The dose was determined by the results of our preliminary experiment and reported human data of the maximum plasma concentrations (*C*_max_) of oseltamivir (115 ng/mL) and Ro 64-0802 (544 ng/mL) [[6]]. Plasma unlabeled oseltamivir and Ro 64-0802 concentrations were measured at 1, 30, and 60 min after [^11^C]oseltamivir injection to confirm the establishment of therapeutic plasma concentration during the PET scan (open arrowheads).

#### Immune activation by systemic poly I:C injection

In the immune activation studies, 8 mg/kg of poly I:C (InvitroGen, Carlsbad, CA, USA) dissolved in sterilized saline was administered intravenously 1 h before the PET scan (gray arrow). To confirm the induction of immune activation, the plasma levels of pro-inflammatory cytokines, IL-6, interleukin 1β (IL-1β), and tumor necrosis factor α (TNF-α) were measured just before the administration of poly I:C, 1 min after the beginning of the PET scan, and at the end of the scan (gray arrowheads).

#### Radiosynthesis of [^11^C]oseltamivir

The radiosynthesis of [^11^C]oseltamivir is described elsewhere [[4]]. The injected radioactivity, specific radioactivity, and mass of [^11^C]oseltamivir at the time of the start of the PET scan were 466 ± 144 MBq, 3.06 ± 1.04 GBq/μmol, and 161 ± 38 nmol (50.4 ± 12.4 μg), respectively.

#### PET and MRI

The PET scans were performed with a SHR-7700 PET camera (Hamamatsu Photonics, Hamamatsu, Japan) in two-dimensional mode, which provides 31 trans-axial slices 3.6 mm (center-to-center) apart, a 33.1-cm field of view (FOV), with an axial FOV of 111.6 mm. The head of the monkey was fixed at the center of the FOV in prone position. A 50-min transmission scan using a ^68^Ge-^68^Ga source for attenuation correction was performed prior to the emission scan. [^11^C]oseltamivir was injected intravenously via the crural vein for 1 min, and a 60-min dynamic scan (36 frames: 12 × 10 s, 6 × 30 s, 5 × 1 min, 5 × 2 min, and 8 × 5 min) was started at the time when the gantry count rate exceeded 100 kcps. The images were reconstructed by filtered back projection method with a Hanning filter, resulting in an in-plane reconstructed resolution of 4.0-mm full width at half maximum.

MRI of the monkey for the preliminary experiment was performed with SIGNA EXCITE HD at 3.0 T (GE Medical Systems, Milwaukee, WI, USA). MR imaging consisted of a short-time inversion recovery sequence (STIR, repetition time (TR) = 5,000 ms, echo time (TE) = 80 ms, inversion time (TI) = 110 ms, FOV = 100 mm, number of slices = 52 to 60, slice thickness = 1 mm without slice gap, 512 × 384 acquisition matrix which after reconstruction was reformatted to a 512 × 512 image matrix, number of excitations = 6, total acquisition time = 72 to 90 min).

#### Arterial blood sampling

Arterial blood samplings were performed for analyzing the time courses of plasma [^11^C]oseltamivir, whole blood ^11^C concentrations, and plasma unlabeled oseltamivir and Ro 64-0802 concentrations. In the immune activation study, the pro-inflammatory cytokine levels were also measured.

In total, 23 arterial blood samples were withdrawn from the femoral artery. The sampling time points after the tracer injection were as follows: every 10 s from 10 s to 1 min, every 15 s from 1 to 3 min, then 3.5, 4, and 5 min, and every 10 min from 10 to 60 min. The blood sample volumes varied from 0.8 to 2 mL, depending on the purpose at each time point.

#### Plasma drug and pro-inflammatory cytokine assay

An aliquot of 0.1-mL plasma was mixed with 2 μg of esterase inhibitor dichlorvos (1 μL of 200 μg/mL in acetonitrile). The samples were stored at −80°C until used. Further sample preparation and LC/MS/MS analysis to determine the concentrations are described elsewhere [[3]]. Area under the concentration-time curve (AUC) between 1 and 60 min was calculated by the trapezoid method.

Arterial blood samples for pro-inflammatory cytokine assay were placed on ice, followed by application of stabilizer (5% *v*/*v*). The plasma was separated by refrigerated centrifugation (20,000 × *g*, 3 min, 4°C) and stored at −80°C until used. Th cytokine levels in plasma were determined using two-site ELISA according to the directions of the manufacturer for monkey IL-6, IL-1β, TNF-α (CKM005, CKM039, CKM009; Cell Sciences, MA, USA). Optical density was measured at 450 nm by microplate reader (Tecan Safire2, TECAN, Zurich, Switzerland). The lower limit of detection for IL-6, IL-1β, and TNF-α was 2 pg/mL each. The test samples and standards were prepared in duplicate.

#### Plasma [^11^C]oseltamivir concentration

After taking arterial blood samplings, the radioactivity of 100 μL of whole blood and 200 μL of plasma samples was measured with a well-type auto-gamma counter (1480 WIZARD 3″ Gamma Counter, Perkin Elmer, Inc., Waltham, MA, USA).

To determine the unmetabolized [^11^C]oseltamivir fraction in plasma, the samples taken at 1, 3, 5, 10, 20, 40, and 60 min were subjected to metabolite analysis by high-performance liquid radiochromatography. An aliquot of 0.5-mL plasma was mixed with the same amount of acetonitrile and then vortexed and centrifuged at 20,000 × *g* at 4°C for 3 min with a refrigerated centrifuge for deproteinization. Then, an aliquot of 0.5 mL of the supernatant was injected into the reverse-phase HPLC system (JASCO Co., Tokyo, Japan). The analytical column used was Waters XBridge OST C18 2.5 μm (10 mm × 50 mm) + Waters Xbridge Prep C18 (10 mm × 10 mm) 5-μm Guard Cartridge, the mobile phase was acetonitrile/50 mM trifluoroacetic acid (22/78) at an isocratic condition, and the flow rate was 8.0 mL/min (retention time 4.2 min). Effluent radioactivity was detected with a homemade NaI(Tl) scintillation detector system [[16]]. The retention time of the radiochromatography peak of [^11^C]oseltamivir was identified by the optical absorption of standard oseltamivir at a detection wavelength of 254 nm. The unmetabolized fraction was calculated as the peak area ratio of unmetabolized [^11^C]oseltamivir to the total peaks detected. Radioactivity recovery in the acetonitrile supernatant, tested in advance, was 91.4%.

The time course of plasma [^11^C]oseltamivir concentrations (plasma input function) was calculated as the product of plasma radioactivity concentration and unmetabolized plasma [^11^C]oseltamivir fraction at each time point. At time points when the unmetabolized fraction was not measured, it was calculated by linear interpolation of the measured fractions.

#### Quantification of plasma-to-brain transfer rate

To estimate the plasma-to-brain transfer rate *K*_1_ (mL/min/cm^3^), the following model was applied. The radioactivity concentration of the brain region of interest (ROI) obtained with PET, *C*_ROI_(t) (kBq/cm^3^), consisted of brain radioactivity concentration *C*_t_(*t*) (kBq/cm^3^) and vascular whole blood radioactivity concentration *C*_w_ (kBq/mL). With the use of the fractional blood volume within the ROI *V*_b_ (mL/cm^3^), *C*_ROI_(*t*) can be expressed as

(1) $$C_{\mathrm{ROI}}\left(t\right)=\left(1-V_{b}\right)C_{t}\left(t\right)+V_{b}C_{w}\left(t\right)$$

Shortly after the intravenous injection, the [^11^C]oseltamivir kinetics in the brain can be described as one-tissue compartment model only with *K*_1_ (mL/min/cm^3^) as follows:

(2) $$\frac{dC_{t}\left(t\right)}{\mathrm{dt}}=K_{1}C_{p}\left(t\right),$$

where *C*_p_ is the plasma input function (kBq/mL). Integration of both sides of Equation 2 and rearrangement with Equation 1 yield:

(3) $$\frac{{C_{\mathrm{RO}}}_{I}\left(t\right)}{C_{w}\left(t\right)}=\left(1-V_{b}\right)K_{1}\frac{\int_{0}^{t}C_{p}\left(s\right)\;\mathrm{ds}}{C_{w}\left(t\right)}+V_{b}$$

**Figure 2:**
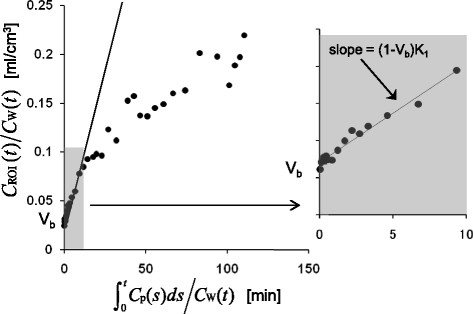
**Integration plot analysis.** Linear regression was performed in the shadowed time segment up to 3.5 min (enlarged to the right).

Then, integration plot analysis [[17]–[19]] was applied to estimate *K*_1_ and *V*_b_ by linear regression up to 3.5 min with Microsoft Excel 2007 (Redmond, WA, USA; Figure 2). The delay of plasma input function was determined by fitting the one-tissue compartment model using the first 10-min data individually. Image and data analyses except for integration plot analysis were performed with PMOD 3.0 (PMOD Technologies, Ltd., Zurich, Switzerland).

#### Statistical analysis

The data are presented individually. Due to the small sample size and gender heterogeneity, statistical test was not performed in this study.

## Results

### Physiological conditions

The mean heart rates during PET scans were 155 to 185 bpm and 130 to 189 bpm for baseline and poly I:C treatment conditions, respectively. Body temperature during poly I:C treatment condition was kept between 36.5°C and 37.4°C.

### Plasma oseltamivir and Ro 64-0802 concentrations

The oseltamivir and Ro 64-0802 concentrations at 1 min after [^11^C]oseltamivir injection, corresponding to the uptake phase, and AUC between 1 and 60 min are shown in Table 1. Both oseltamivir and Ro 64-0802 concentrations were kept at equivalent levels of the reported clinical concentrations at the highest oral dosing [[6]] during the PET scans. Overall, oseltamivir concentrations under poly I:C treatment were lower than those of the baseline condition despite the equivalent dosage, whereas the Ro 64-0802 concentrations were similar.

**Table 1:** Unlabeled drug concentrations in plasma

**Animal**	**Dose (mg/kg)**		**Plasma concentration at 1 min (ng/mL)**				**AUC (1 to 60 min) (ng/mL × min)**			
			**Oseltamivir**		**Ro 64-0802**		**Oseltamivir**		**Ro 64-0802**	
	**Baseline**	**Poly I:C**	**Baseline**	**Poly I:C**	**Baseline**	**Poly I:C**	**Baseline**	**Poly I:C**	**Baseline**	**Poly I:C**
M176	3.4	3.3	590	281	622	494	14,437	10,237	43,252	38,523
F175	3.0	3.0	282	222	749	727	9,291	7,782	48,849	45,976
F174	3.2	3.3	343	190	1,004	1,093	10,667	5,238	65,966	48,924
Mean	3.2	3.2	405	231	792	772	11,465	7,752	52,689	44,474
SD	0.2	0.1	163	46	195	302	2,664	2,500	11,834	5,361

### Plasma pro-inflammatory cytokines

The plasma levels of pro-inflammatory cytokines during the poly I:C treatment scans are listed in Table 2. Although there were no significant elevations of TFN-α and IL-1β, the IL-6 levels of all monkeys were elevated after poly I:C loading. Therefore, the induction of immune activation by poly I:C was confirmed in each of the animals.

**Table 2:** Plasma concentrations of pro-inflammatory cytokines

	**IL-6 [pg/mL]**			**IL-1β [pg/mL]**			**TNF-α [pg/mL]**		
**Animal**	**Baseline**	**1 min**	**60 min**	**Baseline**	**1 min**	**60 min**	**Baseline**	**1 min**	**60 min**
M176	2.74	55.1	739	15.2	12.7	13.3	<2	<2	<2
F175	<2	99.6	525	12.2	7.0	9.3	<2	<2	<2
F174	4.44	14.0	395	51.6	55.1	65.7	<2	<2	<2

### Brain [^11^C]oseltamivir PET imaging

**Figure 3:**
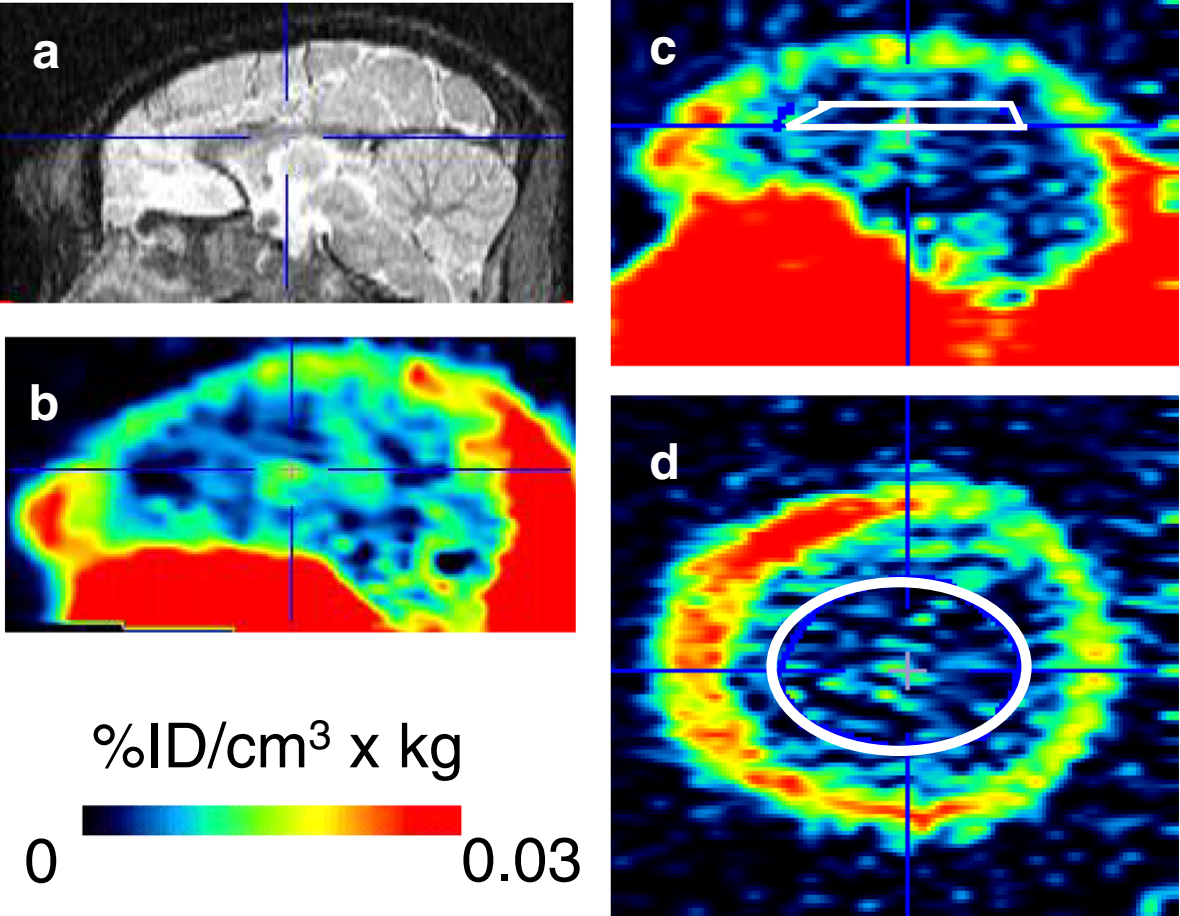
**MR and PET images.** Sagittal planes of MRI **(a)** and the coregistered averaged PET image from 10 to 60 min after [^11^C]oseltamivir injection **(b)** and region of interest **(c**, **d)**.

The PET images of the baseline and poly I:C treatment conditions were similar in all monkeys. Figure 3a shows an MR image of the sagittal plane and the coregistered PET image averaged between 10 and 60 min post injection in the preliminary experiment. Radioactivity concentration was normalized with injected radioactivity dose (ID) and body weight, expressed as%ID/cm^3^ × kg. Radioactivity in the brain was distributed homogeneously and was lower than in surrounding muscle except for a slight accumulation in the ventricle. The coregistered PET image was used as reference for the determination of ROI of the juvenile monkeys whose MR images were unavailable. For the juvenile monkeys, to minimize the radioactivity spillover from outside the brain, ventricle, and major blood vessels, an elliptical ROI was drawn on the central semiovale level of the summed PET image (10 to 60 min) (Figure 3b) to obtain time-activity curves (TACs). The size of ROI was 4.05 ± 1.46 cm^3^ (781 ± 281 voxels) (mean ± SD).

### Plasma [^11^C]oseltamivir kinetics

**Figure 4:**
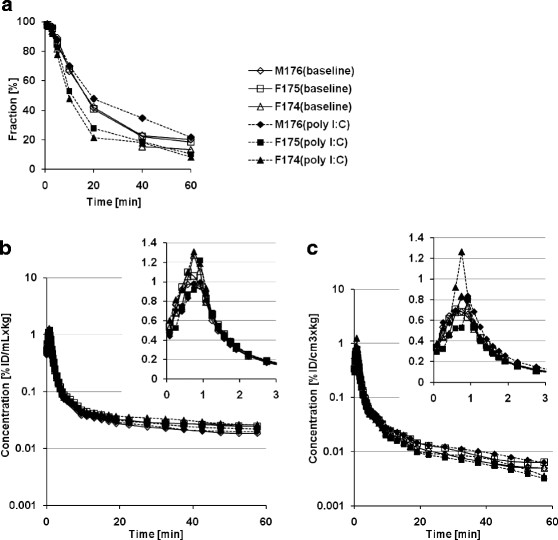
**[**^**11**^**C]oseltamivir kinetics in blood and plasma.** The time course of unmetabolized plasma fraction **(a)**, whole blood TACs **(b)**, and plasma input functions **(c)** in logarithmic scale. The inset graphs correspond to the curves at early times (0 to 3 min). The data are presented individually as baseline (open symbols with solid line) and poly I:C treatment (filled symbols with dashed line) scans.

[^11^C]oseltamivir was gradually metabolized after its intravenous bolus injection as shown in Figure 4a. During the baseline scan of F174, arterial blood sampling was unavailable at 10, 20, and 30 min due to technical troubles. The time courses of the unmetabolized fractions of F175 and M176 were similar under baseline conditions, whereas they fluctuated under the poly I:C treatment. The time courses of normalized whole blood radioactivity concentrations and plasma input functions are shown in Figure 4b,c, respectively. There were no notable differences in normalized whole blood radioactivity concentrations and plasma input functions between baseline and poly I:C treatment conditions.

### Brain time-activity curves

**Figure 5:**
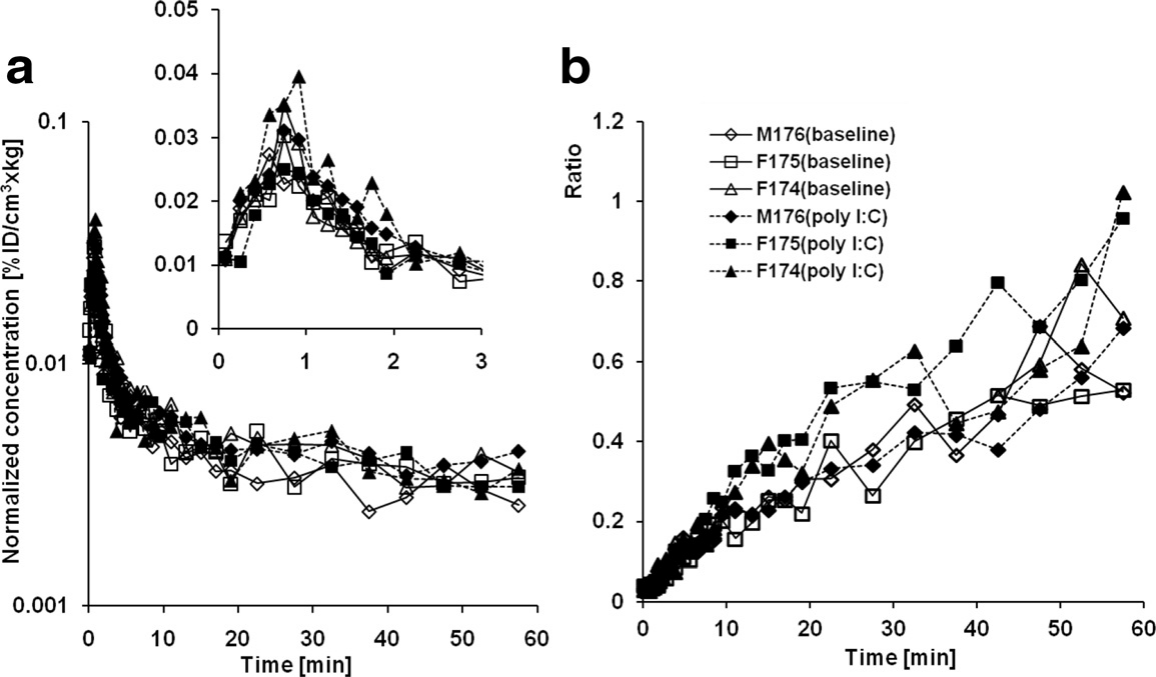
**Brain time-activity curves (TACs). (a)** Brain TACs plotted in logarithmic scale of individual monkey of baseline and poly I:C treatment scans. Brain concentrations normalized with injected radioactivity (ID) and body weight. The inset graph corresponds to TACs at early post-injection times (0 to 3 min). **(b)** The time courses of brain-to-plasma radioactivity concentration ratio are plotted individually. The symbols and lines correspond to individual animals and conditions as in Figure 4.

**Figure 6:**
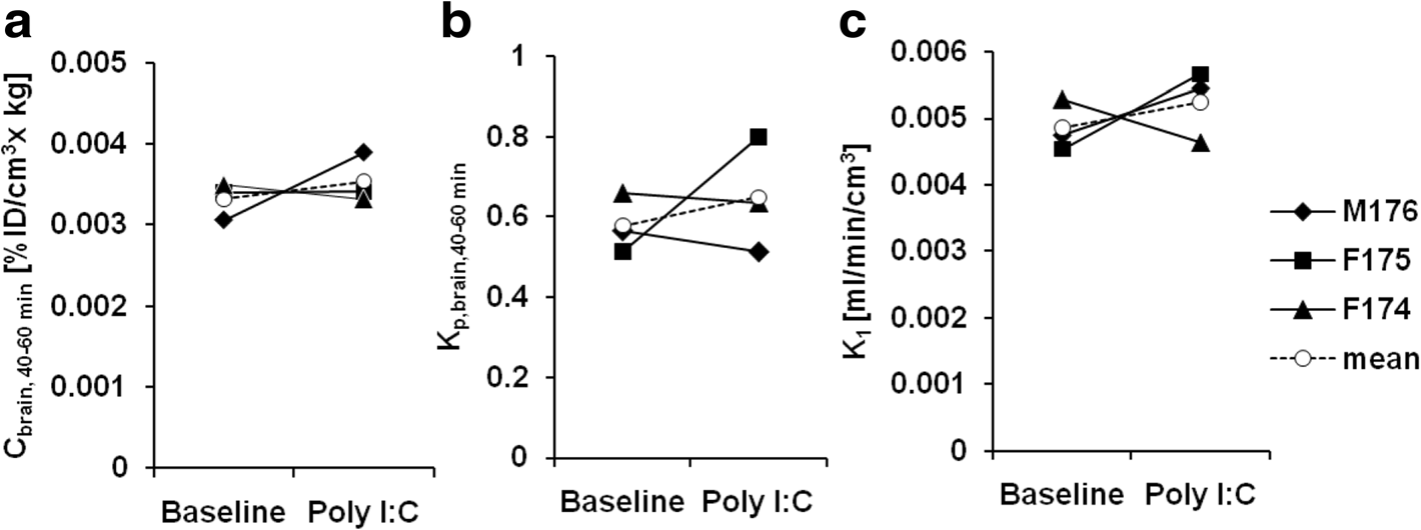
**The changes of brain [**^**11**^**C]oseltamivir uptake by poly I:C. (a)** Normalized brain concentration, **(b)** brain-to-plasma [^11^C]oseltamivir concentration ratio, and **(c)** plasma-to-brain transfer rate (*K*_1_) of individual monkeys (solid lines) and mean (dashed lines) are shown.

The individual TACs of the monkey brains are shown in Figure 5a. The effect of poly I:C treatment on the brain radioactivity concentrations was quantitatively compared using average normalized concentrations between 40 and 60 min (*C*_brain,40-60min_) as shown in Figure 6a. The mean *C*_brain,40-60min_ values in the monkeys were 0.0033 ± 0.0002% ID/cm^3^ × kg and 0.0035 ± 0.0003% ID/cm^3^ × kg for baseline and after poly I:C treatment, respectively. The effect of the poly I:C treatment on *C*_brain,40-60min_ was not notable.

The time courses of the plasma-to-brain concentration ratio (*K*_p,brain_) were plotted in Figure 5b. The ratio between 40 and 60 min (*K*_p,brain,40-60min_) was calculated, and the changes by poly I:C treatment are shown in Figure 6b. The mean *K*_p,brain,40-60min_ values of the three monkeys were 0.58 ± 0.07 for baseline and 0.65 ± 0.14 for poly I:C treatment conditions. The effect of poly I:C treatment on *K*_p,brain,40-60min_ was not notable.

### Plasma-to-brain [^11^C]oseltamivir transfer rate

A typical example of the integration plot is shown in Figure 2. The linear segment in the first few minutes confirmed a negligible efflux from the brain within the period. The mean *K*_1_ values of the three monkeys were 0.0049 ± 0.0004 mL/min/cm^3^ for baseline and 0.0053 ± 0.0005 mL/min/cm^3^ for the poly I:C treatment. The *K*_1_ values at baseline and after poly I:C treatment are compared in Figure 6c. *K*_1_ did not notably increase by poly I:C treatment. The estimated *V*_b_ ranged from 0.022 to 0.028 mL/cm^3^. This value was comparable to the reported cerebral blood volume in monkeys of 0.035 mL/cm^3^ [[20]].

## Discussion

In the present study, we examined the effect of innate immune activation on the CNS uptake of oseltamivir at its clinically relevant plasma concentrations using [^11^C]oseltamivir and PET in living juvenile monkeys. We used intravenous poly I:C administration to activate the immune response to simulate viral infection. Poly I:C stimulates toll-like receptor 3 (TLR3), one of the innate immune-recognition receptors, whereas influenza virus is recognized by TLR7. Both TLRs are expressed in endosome, and their stimulations result in the induction of pro-inflammatory cytokines via different signaling pathways [[21]]. This study demonstrated an immediate increase of plasma IL-6 after poly I:C treatment, while no alterations were observed in TNF-α and IL-1β in the first 2 h (Table 2). The elevation of plasma IL-6 has been reported as the common feature in influenza patients including children [[22],[23]], and therefore, the immune responses to poly I:C and influenza virus are similar despite differences in the signaling pathways. From the rapid elevation of plasma IL-6 throughout the experimental period, the immune activation model in this study was considered to simulate a part of the innate immune response to influenza virus infection. The lack of elevation of body temperature is considered to be due to the hypothermic effect of the applied anesthesia.

In the quantification of the CNS concentration of low BBB permeable radiolabeled ligand, the linearity of measured radioactivity concentration from a low level and the contribution of intravascular radioactivity must be considered significant. The lowest decay-uncorrected radioactivity concentration in the brain was approximately 0.2 kBq/cm^3^. The PET scanner used in this study has a linearity between 0.05 and 50 kBq/cm^3^. Therefore, the brain concentration was accordingly quantified with the PET scanner. Nevertheless, in consideration of the noise, we placed ROI on the central semiovale where the largest ROI was available for better statistics. Regarding intravascular radioactivity, both the TACs of blood and brain peaked at about 1 min simultaneously, as shown in Figures 4b and 5a. At this point, the peak of blood radioactivity was approximately 1% ID/mL × kg (Figure 4b). Using *V*_b_ of 0.025 mL/cm^3^ estimated by integration plot analysis, the vascular radioactivity at the peak was estimated to be about 0.025% ID/cm^3^ × kg. This value is close to the peak values of the brain TACs (Figure 5a). At later times, between 40 and 60 min post injection, the mean normalized blood concentration of poly I:C treatment condition was 0.025% ID/mL × kg. Using *V*_b_ of 0.025 mL/cm^3^, the vascular radioactivity in the brain ROI can be estimated as 0.000625% ID/cm^3^ × kg, corresponding to 18% of *C*_brain,40-60min_ (0.0035% ID/cm^3^ × kg). This indicates that the contribution of vascular radioactivity at later times became less than in the first few minutes. To remove the contribution of intravascular radioactivity from the measured brain concentration, Equation 1 can be applied to estimate the net brain concentration *C*_t_(*t*) using *V*_b_ obtained from integration plot analysis (Equation 3). By this intravascular radioactivity correction (CBV-correction), *C*_brain,40-60min_ and *K*_p, brain_ overall became 15% less than the uncorrected values, but the changes of *C*_brain,40-60min_ and *K*_p,brain_ values by poly I:C treatment were very similar. The individual CBV-corrected results are shown in Additional file 1.

With respect to the plasma-to-brain transfer rate, the estimated *K*_1_ values were considerably lower than monkey CBF, which is approximately 0.5 mL/min/g [[20]], and therefore, the extraction fraction (*E*) is approximated to 0.01 using the relationship *K*_1_ = *E*∙CBF. According to the Crone-Renkin equation [[24],[25]], the penetration of [^11^C]oseltamivir across the BBB is in a diffusion-limited manner, and therefore, *K*_1_ is not considered to reflect CBF.

The CNS radioactivity concentration measured with PET includes both [^11^C]oseltamivir and [^11^C]Ro 64-0802. The latter is less BBB-permeable than oseltamivir, so the radioactive metabolite [^11^C]Ro 64-0802 penetration into the CNS is less likely. But, the fact that the immunoreactivity of CES1 was detected in human brain endothelial cells [[26]] suggests that [^11^C]Ro 64-0802 can be formed from [^11^C]oseltamivir in brain capillary endothelial cells. Although Oat3 and Mrp4 facilitate removal of Ro 64-0802, which is formed in the endothelial cells from the brain to the blood [[5]], part of the radioactivity in the brain may be attributed to [^11^C]Ro 64-0802, but the major portion of CNS radioactivity can be ascribed to [^11^C]oseltamivir.

With the use of CNS concentration measured with PET, one can roughly estimate the CNS drug concentration under therapeutic dose. Using *C*_brain,40-60min_ of 0.0035% ID/cm^3^ × kg, the CNS oseltamivir concentration at a therapeutic dose of 2 mg/kg can be approximated as 0.22 μM. Or, with the use of plasma-to-brain ratio *K*_p,brain,40-60min_ and the reported *C*_max_ in a clinical study of 115 ng/mL [[6]], the CNS concentration can be estimated as high as 0.37 μM. It has been reported that no inhibitory effect against recombinant human neuramidases was detected up to 1 mM of both oseltamivir and Ro 64-0802 [[24]]. This report also demonstrated that no relevant inhibitory effect against various molecular targets of neurotransmitter system was observed up to 3 μM for both oseltamivir and Ro 64-0802 [[27]]. Alternatively, a previous study of electrophysiology experiments using mouse brain reported that the pharmacological effect was detected at ED_50_ of 10.2 μM for oseltamivir and 0.7 μM for Ro 64-0802 [[28]]. The CNS concentrations estimated with the PET data are lower than these reported concentrations.

The normalized brain concentration at later times and the transfer rate (*K*_1_ or CL_uptake, brain_) were about half of those of a previous report using adolescent monkeys [[8]]. Several factors including different anesthesia can be considered. First, the monkeys used in this study were in a later developmental stage with almost double the body weight of those used in the previous report. Therefore, the different developmental stage would be a reason for the difference. Second, the values of the parameters are considered to depend on the placing of the ROI. We chose the central semiovale. As described previously, the radioactivity was considered relatively lower than in other regions. Third, the number of blood samples for input function used for integration plot analysis can be a factor that causes different results in calculated transfer rates. In this study, we took samples 12 times, with the shortest interval being 10 s, between 10 s and 2.5 min, whereas four samples with the shortest interval of 30 s were collected between 30 s and 2.5 min in the previous study.

One of the limitations of this study was the small sample size and gender heterogeneity. For this reason, statistical evaluation of changes in oseltamivir brain uptake by poly I:C treatment could not be strictly performed.

There have been no studies to test the alterations of a drug penetration into the CNS by immune activation in vivo in nonhuman primates under therapeutic dose. PET imaging of radiolabeled drug in a monkey model of immune activation is advantageous for assessing the changes in drug distribution by viral infection directly in vivo.

## Conclusions

[^11^C]oseltamivir uptake into the CNS in a juvenile monkey model did not show notable change by immune activation induced by poly I:C administration under clinically relevant plasma levels of oseltamivir and its active metabolite Ro 64-0802.

## Competing interests

The authors declare that they have no competing interests.

## Authors’ contributions

CS, AON, YN, TM, SO, MI, and TS conceived of and contributed to the study design. CS, AON, YN, and SO performed the experiments and acquired the PET data. CS analyzed the data and drafted the manuscript. CS, AON, YN, and TM participated in interpretation of the data. AON, MRZ, MH, and HK revised the manuscript. MT, KF, TI, and MRZ carried out the radiochemical synthesis and radio-HPLC metabolite analysis. SI and HK quantified the plasma unlabeled drug concentrations. HI and YS reviewed the manuscript. TS supervised the study. All authors read and approved the final manuscript.

## Additional file

## Supplementary Material

Additional file 1 **The changes of brain [**^**11**^**C]oseltamivir uptake by poly I:C after CBV-correction.** Click here for file
