# Supplementary material for: Evaluation of [11C]oseltamivir uptake into the brain during immune activation by systemic polyinosine-polycytidylic acid injection: a quantitative PET study using juvenile monkey models of viral infection
Source: EJNMMI Res. 2014 Jul 2;4:24. doi: 10.1186/s13550-014-0024-8 (PMC4100568; doi:10.1186/s13550-014-0024-8)
Supplement: Additional file 1 — The changes of brain [ 11 C]oseltamivir uptake by poly I:C after CBV-correction. [file s13550-014-0024-8-S1.pdf]

Additional file 1.

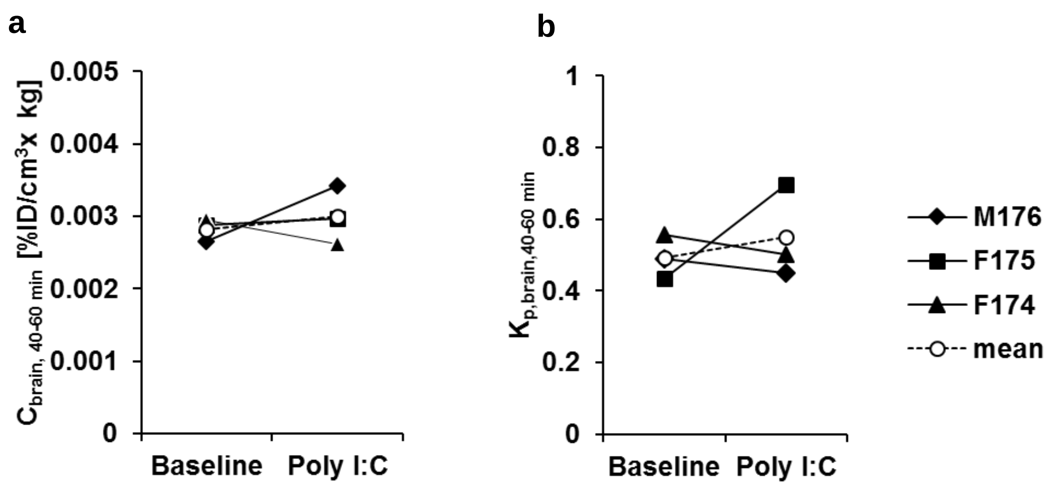

The changes of brain [^{11}C]oseltamivir uptake by poly I:C after CBV-correction. CBV-corrected normalized brain concentration (a) and brain to plasma and [^{11}C]oseltamivir concentration ratio (b) of individual monkeys (solid lines) and mean (dashed lines) are shown.
